# Special Issue “Cells and Molecules in Bone Remodeling and Repair”

**DOI:** 10.3390/ijms262411796

**Published:** 2025-12-06

**Authors:** Jung-Eun Kim

**Affiliations:** 1 Department of Molecular Medicine, School of Medicine, Kyungpook National University, Daegu 41944, Republic of Korea; kjeun@knu.ac.kr; Tel.: +82-53-420-4949; 2 BK21 FOUR KNU Convergence Educational Program of Biomedical Sciences for Creative Future Talents, Department of Biomedical Science, Kyungpook National University, Daegu 41944, Republic of Korea; 3 Cell and Matrix Research Institute, Kyungpook National University, Daegu 41944, Republic of Korea

Bone is a highly dynamic tissue that undergoes continuous remodeling from birth throughout life, replacing aged bone tissue with newly formed bone to maintain structural integrity. In addition, bone possesses a remarkable capacity for repair, orchestrated by complex and well-coordinated physiological processes during micro-crack healing and fracture repair. Bone remodeling and repair are tightly regulated by the interplay between bone cells and the diverse molecular factors that shape the bone microenvironment.

This Special Issue of the *International Journal of Molecular Sciences*, titled *“Cells and Molecules in Bone Remodeling and Repair”* brings together recent advances that expand our understanding of the molecular and cellular mechanisms governing bone physiology. It features seven contributions, including three comprehensive reviews and four original research papers, covering diverse topics such as mechanotransduction, molecular signaling, regenerative therapies, and bioengineering approaches. Together, these works provide novel insights into the fundamental processes of bone remodeling and repair while pointing toward innovative strategies for therapeutic application.

Berni et al. presented a systematic review on the efficacy of low-level laser therapy (LLLT) in promoting bone healing, emphasizing the need to elucidate the underlying molecular mechanisms and the influence of treatment parameters such as wavelength, energy density, and cell type [1]. Zhang et al. reviewed the cell-specific role of SHP2, a protein tyrosine phosphatase encoded by *PTPN11*, in bone homeostasis and remodeling, highlighting the dual nature of SHP2 signaling across osteoblasts, osteoclasts, and mesenchymal stem cells and its therapeutic potential [2]. Gargalionis et al. explored the interplay between Runx2 and polycystins in bone mechanotransduction, underscoring the central role of mechanical forces in bone physiology and their mechanotherapeutic implications [3]. Collectively, these reviews illuminate the multifaceted regulation of bone tissue—from biophysical stimulation and mechanical sensing to cell-specific signaling pathways—providing a foundation for developing targeted therapeutic interventions.

García-Sánchez et al. demonstrated that the transient silencing of secreted frizzled-related protein 1 (SFRP1) in mesenchymal stem cells (MSCs) generates a pro-osteogenic secretome enriched with osteogenesis-related proteins, thereby enhancing the regenerative capacity of MSC-derived secretomes [4]. Castoldi et al. provided a transcriptomic profile of primary human osteoblast-like cells cultured on a 3D trabecular titanium scaffold, revealing gene expression patterns linked to osteogenic differentiation and osseointegration [5]. Both studies apply omics approaches to uncover molecular mechanisms that can be harnessed to optimize bone regeneration, implant integration, and therapeutic strategies.

Silva et al. investigated the dose–response effects of photobiomodulation (PBM) on the osteogenic differentiation of adipose-derived stem cells in 3D hydrogel cultures [6]. Their findings highlight the importance of light parameters in modulating stem cell behavior and support PBM, particularly in combination with biomaterial scaffolds, as a promising strategy for bone regeneration. Zhang et al. uncovered an epigenetic mechanism whereby protein arginine methyltransferase 7 (PRMT7) activates PTEN to promote bone regeneration in female mice, revealing a sex-specific regulatory axis with significant implications for skeletal repair [7]. Together, these studies underscore how biophysical stimulation, engineered culture systems, and molecular targeting can be integrated to develop innovative MSC-based treatments for osteoporosis and other degenerative bone diseases.

As Guest Editor of this Special Issue, dedicated to the role cells and molecules in bone remodeling and repair, I am deeply grateful to all contributing authors for their valuable work and commitment to advancing the field. Collectively, these studies provide meaningful insights into the cellular and molecular foundations of bone remodeling and repair, while also highlighting new opportunities for regenerative therapies and targeted treatments. Looking ahead, continued research will be essential to fully elucidate the molecular mechanisms underlying bone diseases and to translate these findings into effective clinical applications. It is my sincere hope that this Special Issue will inspire further investigation and foster innovative approaches for the prevention and treatment of bone disorders.
